# Case Report: Postpartum diagnosis and microsurgical management of a cervicothoracic intramedullary spinal ependymoma clinically manifesting during the third trimester of pregnancy

**DOI:** 10.3389/fonc.2026.1926208

**Published:** 2026-09-08

**Authors:** Talgat T. Kerimbayev, Daryn Borangaliyev, Azamat Abenov, Daniyar K. Zhamoldin, Galymzhan Kadirbekov, Zhandos Tuigynov, Yergen Kenzhegulov, Viktor G. Aleinikov, Berik Zhetpisbayev, Yermek Urunbayev, Nurzhan Abishev, Meirzhan Oshayev, Aisa Z. Nurperisov, Zhanibek Baiturlin, Makar Solodovnikov, Serik K. Akshulakov

**Affiliations:** 1 Department of Spinal Neurosurgery and Peripheral Nervous System Pathology, Joint Stock Company (JSC) “National Centre for Neurosurgery”, Astana, Kazakhstan; 2 Department of Minimally Invasive Neurosurgery, Joint Stock Company Joint Stock Company (JSC) “National Centre for Neurosurgery”, Astana, Kazakhstan; 3 Joint Stock Company (JSC) “National Centre for Neurosurgery”, Astana, Kazakhstan

**Keywords:** intramedullary spinal cord tumour, microsurgical resection, postpartum period, pregnancy, spinal ependymoma

## Abstract

**Background:**

Intramedullary spinal cord ependymomas are the most common intramedullary tumours in adults. However, clinical manifestation during pregnancy is exceptionally rare, and diagnosis may be delayed because pain, sensory disturbances, and muscle weakness are often mistakenly attributed to the common musculoskeletal changes associated with pregnancy.

**Case description:**

A 29-year-old multiparous woman developed progressive back pain, numbness, and weakness in both lower extremities at 30 weeks of gestation. She was initially managed conservatively. Following an uncomplicated spontaneous delivery at 38 weeks of gestation, her neurological condition deteriorated rapidly, with the development of severe spastic paraparesis, sensory impairment below the T1 level, urinary retention, and constipation. Magnetic resonance imaging revealed a centrally located contrast-enhancing intramedullary tumour extending from C7 to T5, accompanied by fusiform expansion of the spinal cord. On postpartum day 38, the patient underwent C7–T5 laminectomy, midline myelotomy, and microsurgical gross total resection of the tumour under multimodal intraoperative neurophysiological monitoring. Despite intraoperative deterioration of left-sided motor responses, no postoperative worsening of the neurological deficit or other surgery-related complications were observed. Following multidisciplinary tumour board review, adjuvant proton beam therapy was delivered to the tumour bed at a total dose of 54 Gy. Сontrast follow-up MRI performed at 6 months showed no convincing evidence of disease progression; Initial histopathological examination classified the tumour as an ependymoma, NOS, CNS WHO grade 3. Molecular testing revealed no mutations in the IDH1 or IDH2 genes; a heterozygous deletion of the MYB gene was additionally identified. The tumour specimen was referred for further specialist neuropathological review. At 6-month follow-up, lower-extremity muscle strength had improved from Medical Research Council grade 2/5 to 3–4/5, functional status had improved from grade IV to grade III on the modified McCormick scale, and partial recovery of bladder function was observed. No clinical or radiological evidence of disease progression was identified.

**Conclusion:**

The development of progressive motor deficit, a defined sensory level, pyramidal signs, or sphincter dysfunction during pregnancy or the postpartum period warrants urgent neurological assessment and MRI of the spine and spinal cord.

## Introduction

Primary intramedullary spinal cord tumours are rare neoplasms, accounting for approximately 2–4% of all central nervous system tumours and 5–10% of spinal tumours (1). In adults, ependymomas are the most common histological type of intramedullary tumour, accounting for approximately 60–70% of these neoplasms (2). Despite the relatively slow growth of most spinal ependymomas, delayed diagnosis may result in severe myelopathy, sphincter dysfunction, and permanent disability. Functional outcomes therefore depend largely on the patient’s preoperative neurological status and the timely provision of surgical treatment (3).

Central nervous system tumours first diagnosed during pregnancy are rare, with a reported incidence of approximately 3–6 cases per 100,000 pregnancies (4). Most pregnancy-associated neoplasms described in the literature are intracranial tumours, whereas information on intramedullary spinal cord tumours occurring during pregnancy is limited to isolated case reports and small literature reviews (5, 6). The diagnosis of these tumours may be challenging because back pain is common during pregnancy, and early neurological symptoms may be mistakenly interpreted as physiological manifestations of gestation (7–9). However, progressive muscle weakness, a defined sensory level, pyramidal signs, or bladder dysfunction are not physiological manifestations of pregnancy and warrant urgent neurological assessment and MRI of the spine and spinal cord (6, 10).

We present a rare case of a long-segment cervicothoracic intramedullary ependymoma that became clinically manifest during the third trimester of pregnancy and was diagnosed in the postpartum period by non-contrast MRI following rapid neurological deterioration. Distinctive features of this case include symptom onset during late pregnancy, rapid postpartum neurological deterioration, extensive involvement of the cervicothoracic spinal cord, and gross total tumour resection without intraoperative deterioration in neurophysiological parameters.

## Clinical presentation

A previously healthy 29-year-old woman, gravida 7 para 5 (G7P5), developed progressive numbness in both lower extremities, leg weakness, and persistent back pain at approximately 30 weeks of gestation. According to the patient, these symptoms were initially attributed to pregnancy; consequently, she received conservative treatment without undergoing a comprehensive neurological assessment or spinal MRI. The pregnancy was otherwise uncomplicated and culminated in a spontaneous vaginal delivery at 38 weeks of gestation on 2 November 2025.

The neurological symptoms did not resolve after delivery. Over the following several weeks, the lower-extremity weakness progressively worsened. The patient became unable to stand independently and could walk only with assistance. She also developed severe cervicothoracic pain, progressive sensory impairment below the T1 level, urinary retention with loss of bladder-filling sensation requiring catheterisation, and constipation.

Because of the rapidly progressive myelopathy, non-contrast MRI of the cervical and thoracic spine was performed at a regional medical institution on 2 December 2025. The examination revealed a long-segment intramedullary lesion extending from C7 to T5, accompanied by fusiform expansion of the spinal cord. On 8 December 2025, the patient was referred to our specialised neurosurgical centre for further evaluation and treatment. The chronological sequence of the patient’s clinical presentation, diagnostic evaluation, treatment, and follow-up is summarized in **Table 1**.

**Table 1:** Clinical timeline of the episode of care.

Time point	Clinical event	Management/findings
Approximately 30 weeks of gestation	Onset of persistent back pain, bilateral lower-extremity numbness, and progressive leg weakness	Symptoms were initially attributed to pregnancy; conservative treatment was provided without comprehensive neurological assessment or spinal MRI
38 weeks of gestation — 2 November 2025	Uncomplicated spontaneous vaginal delivery	No neurological improvement after delivery
4 November 2025	Discharge from the maternity hospital	Patient’s general condition was considered stable; neurological symptoms persisted
Postpartum weeks 1–5	Progressive neurological deterioration	Increasing lower-extremity weakness, inability to stand independently, assisted ambulation, severe cervicothoracic pain, sensory impairment below T1, urinary retention with loss of bladder-filling sensation requiring catheterisation, and constipation
2 December 2025	Non-contrast MRI of the cervical and thoracic spine at a regional medical institution	Long-segment intramedullary lesion extending from C7 to T5, with fusiform expansion of the spinal cord
5 December 2025	Discharge from the regional medical institution	Patient’s condition remained stable; referral to a specialised neurosurgical centre was recommended
Postpartum day 36 — 8 December 2025	Admission to the tertiary neurosurgical centre	Severe myelopathy; bilateral lower-extremity strength of MRC grade 2/5; sensory impairment below T1; urinary retention; modified McCormick grade IV
8 December 2025	Multidisciplinary clinical assessment	Surgical treatment was recommended because of rapidly progressive myelopathy and the extensive intramedullary lesion
10 December 2025	Surgery	C7–T5 laminectomy, midline myelotomy, and microsurgical gross total resection under continuous intraoperative neurophysiological monitoring
Postoperative day 1 — 11 December 2025	Early postoperative MRI	Postoperative resection cavity with no visible residual tumour
18 December 2025	Initial histopathological examination	Ependymoma, NOS, CNS WHO grade 3
1 January 2026	Discharge from the tertiary neurosurgical centre	Persistent myelopathy with early gradual motor improvement; no cerebrospinal fluid leakage, wound infection, or other surgery-related complications
26 January 2026	Multidisciplinary tumour board review	Adjuvant proton beam therapy to the postoperative tumour bed was recommended on the basis of the initial CNS WHO grade 3 diagnosis and the long-segment C7–T5 involvement
28 January–12 March 2026	Adjuvant proton beam therapy	Image-guided proton therapy delivered to the postoperative tumour bed at 1.8 Gy per fraction to a total dose of 54 Gy
Approximately 6 months postoperatively — 10 June 2026	Clinical and radiological follow-up	Lower-extremity strength improved to MRC grade 3–4/5; modified McCormick grade improved from IV to III; partial recovery of bladder function. Сontrast MRI showed no convincing evidence of disease progression; contrast-enhanced follow-up MRI was recommended

## Diagnostic assessment

On admission, 36 days after delivery, the patient was fully conscious, with a Glasgow Coma Scale score of 15. Cranial nerve function was intact, and no motor deficits were identified in the upper extremities. Neurological examination of the lower extremities revealed severe spastic paraparesis, with muscle strength predominantly graded as 2/5 on the Medical Research Council scale, increased muscle tone of the spastic type, bilateral hyperreflexia, and pathological pyramidal signs. Sensory impairment below the T1 level and urinary retention were also present. Because of the severe motor deficit, the patient was unable to stand or walk independently; therefore, gait assessment and the Romberg test could not be performed. Her functional status corresponded to grade IV on the modified McCormick scale.

**Figure 1:**
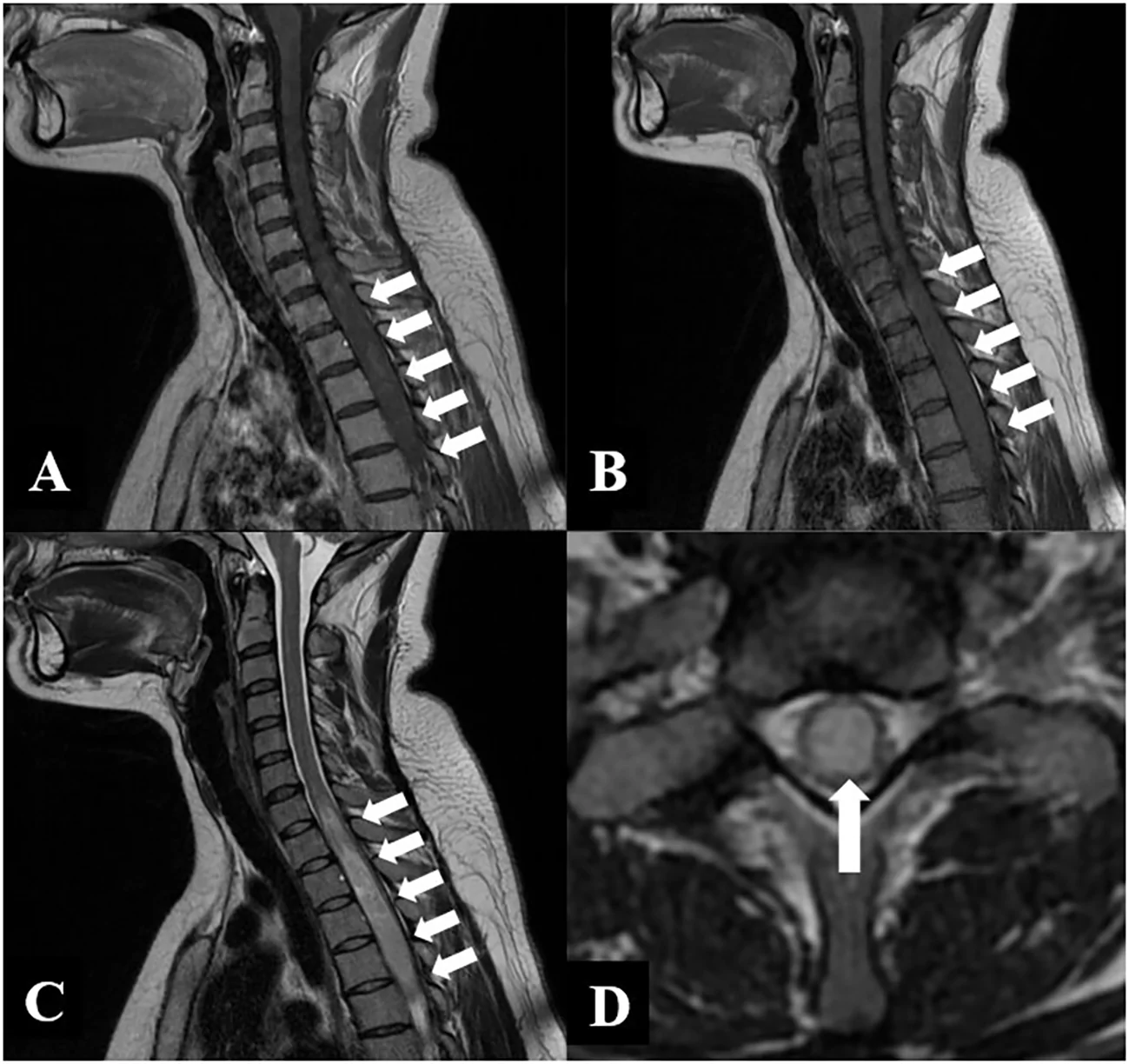
Preoperative magnetic resonance imaging of the cervicothoracic spinal cord. **(A)** Sagittal contrast-enhanced T1-weighted image; **(B)** Sagittal T1-weighted image; **(C)** Sagittal T2-weighted image; **(D)** Axial contrast-enhanced T1-weighted image. Images demonstrate a centrally located long-segment intramedullary tumour extending from C7 to T5, with fusiform spinal cord expansion and contrast enhancement. White arrows indicate the tumour.

For preoperative planning, contrast-enhanced MRI of the cervical and thoracic spine was repeated at our institution after the initial non-contrast examination performed at the regional medical institution (**Figure 1**). The principal differential diagnoses included intramedullary ependymoma, astrocytoma, haemangioblastoma, and an inflammatory demyelinating lesion of the spinal cord.

Ependymoma was considered the most likely diagnosis because the tumour was centrally located, caused symmetrical fusiform expansion of the spinal cord, demonstrated contrast enhancement, and had relatively well-defined margins. Astrocytoma was considered less likely because of the absence of an infiltrative growth pattern, eccentric location, and asymmetrical cord expansion. Haemangioblastoma was also considered unlikely because no distinct mural nodule, prominent flow voids, exophytic hypervascular component, or marked associated syringomyelia was identified.

The gradual progression of neurological deficits, the presence of a persistent contrast-enhancing mass causing spinal cord expansion, and the absence of clinical evidence of an inflammatory disorder argued against longitudinally extensive transverse myelitis and tumefactive demyelination. Because contrast-enhanced MRI was performed after delivery, the foetus was not exposed to a gadolinium-based contrast agent.

## Therapeutic intervention

The patient underwent microsurgical tumour resection under general anaesthesia with continuous multimodal intraoperative neurophysiological monitoring, including motor evoked potentials (MEPs) and somatosensory evoked potentials (SSEPs). After prone positioning, a standard posterior midline approach was performed. Following exposure of the posterior elements, C7–T5 laminectomy was completed. The dura mater was opened through a midline durotomy under the operating microscope and suspended laterally.

**Figure 2:**
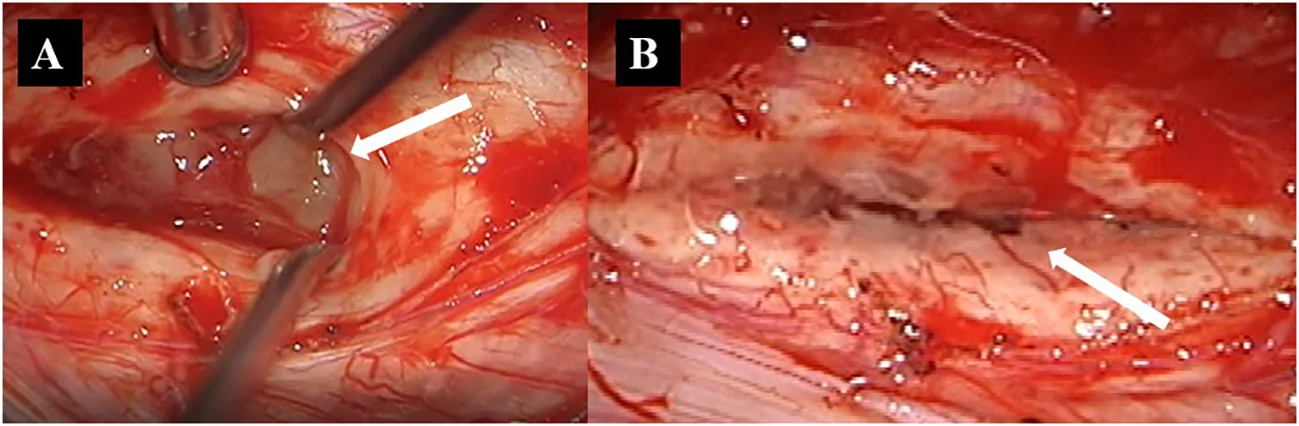
Intraoperative photographs during microsurgical resection. **(A)** The intramedullary tumour is exposed after midline myelotomy. **(B)** Surgical field after gross total tumour resection, showing the resection cavity.

After identification of the posterior median sulcus, a meticulous midline myelotomy was performed to access the intramedullary lesion while minimizing dorsal column injury. A greyish, relatively firm tumour was encountered. The lesion appeared well demarcated from the adjacent spinal cord parenchyma, allowing development of a microsurgical dissection plane. Circumferential sharp and blunt dissection was performed while preserving neural tissue and pial vessels whenever possible. The tumour was progressively mobilized and removed, achieving gross total resection. Restoration of spinal cord pulsation was observed after tumour removal. During tumour resection, motor responses remained stable in the right upper and lower extremities, whereas responses from the left upper extremity became markedly reduced and reproducible responses from the left lower extremity were absent by the end of the procedure. Despite these intraoperative neurophysiological changes, no postoperative worsening of the neurological deficit was observed. (**Figure 2**).

## Histopathological and molecular findings

**Figure 3:**
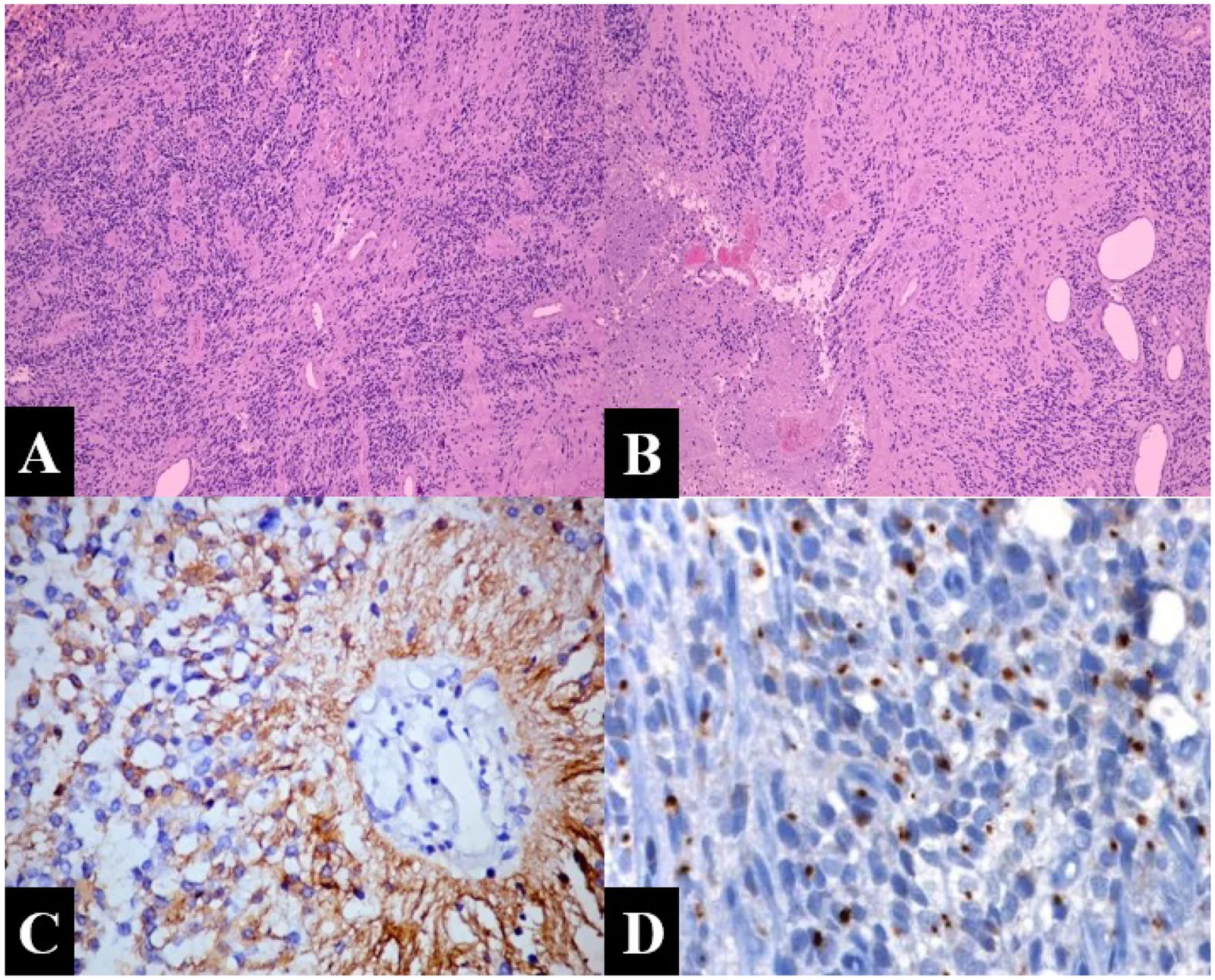
Histopathological and immunohistochemical findings of the resected spinal ependymoma. **(A)** Haematoxylin and eosin staining showing the general tumour morphology with perivascular pseudorosette formation (×100). **(B)** Haematoxylin and eosin staining demonstrating focal tumour necrosis (×100). **(C)** Immunohistochemical staining showing GFAP expression in tumour cells (×200). **(D)** EMA immunohistochemical staining demonstrating characteristic dot-like cytoplasmic immunoreactivity (×200).

On gross examination, the surgical specimen consisted of multiple fragments of soft, greyish-pink tissue measuring up to 6 × 1.5 × 1 cm. On initial microscopic examination, the tumour was composed of moderately pleomorphic round-to-polygonal cells with hyperchromatic nuclei. Numerous perivascular pseudorosettes with perivascular anuclear fibrillary zones were identified. Increased mitotic activity, microvascular proliferation, focal coagulative necrosis, intratumoral haemorrhage, and focal calcifications were also described. Immunohistochemical examination demonstrated GFAP expression and characteristic dot-like cytoplasmic EMA immunoreactivity, supporting the ependymal differentiation of the tumour (**Figure 3**).

According to the initial histopathological examination dated 18 December 2025, the tumour was classified as an ependymoma, NOS, CNS WHO grade 3. The original pathology report described increased mitotic activity but did not provide a quantitative mitotic count. A Ki-67 proliferation index was not available. Subsequent molecular genetic testing detected no mutations in IDH1 or IDH2 and identified a heterozygous deletion of MYB. These findings did not permit assignment of the tumour to a specific molecular type of ependymoma according to the WHO Classification of Tumours of the Central Nervous System, fifth edition (WHO CNS5). The specimen was therefore referred for additional specialist neuropathological review (**Figure 3**).

## Follow-up and outcomes

The early postoperative course was uneventful. The patient continued to exhibit manifestations of myelopathy, including spastic paraparesis of the lower extremities, sensory impairment below the T1 level, and neurogenic bladder dysfunction characterised by urinary retention. Nevertheless, lower-extremity motor function gradually improved, with no postoperative worsening of the neurological deficit. The patient was mobilised, was able to sit independently, and moved around the ward using a wheelchair. Because urinary retention persisted, the urinary catheter remained in place at the time of discharge. No cerebrospinal fluid leakage, surgical-site infection, or other postoperative complications were recorded.

**Figure 4:**
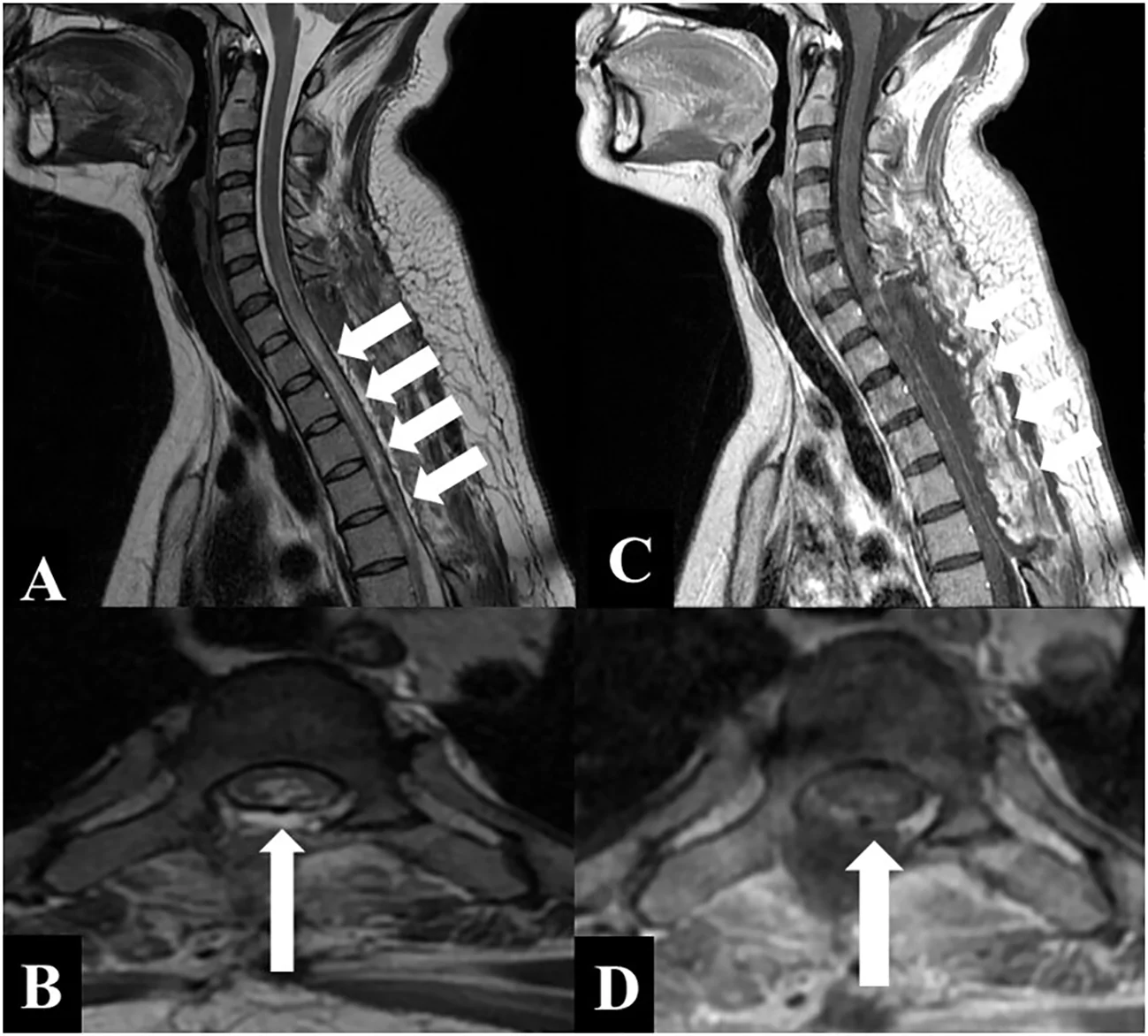
Postoperative 1-day magnetic resonance imaging of the cervicothoracic spinal cord. **(A)** Sagittal T2-weighted image; **(B)** Axial T2-weighted image; **(C)** Sagittal contrast-enhanced T1-weighted image; **(D)** Axial contrast-enhanced T1-weighted image. Images demonstrate postoperative changes following gross total resection, including a postoperative CSF-filled resection cavity without evidence of residual enhancing tumour. White arrows indicate the postoperative resection cavity (site of tumour removal).

Early contrast-enhanced postoperative MRI demonstrated no residual contrast-enhancing tumour, consistent with gross total resection (**Figure 4**). The patient was discharged in a stable condition for outpatient care on postoperative day 21. Continued neurological rehabilitation and referral to a specialised oncology centre for determination of further management and imaging surveillance were recommended.

## Adjuvant treatment

Following discharge, the patient was referred to the National Scientific Cancer Centre to determine further management. The case was reviewed by a multidisciplinary tumour board on 26 January 2026 (Tumour Board Protocol No. 263). Based on the pathological diagnosis of ependymoma, NOS, CNS WHO grade 3, the board recommended adjuvant proton beam therapy to the postoperative tumour bed. The tumour location and its considerable craniocaudal extent were additionally considered during treatment planning.

Between 28 January and 12 March 2026, the patient completed a full course of adjuvant image-guided proton beam therapy using the ProBeam system. A dose of 1.8 Gy per fraction was delivered to a total dose of 54 Gy. Pretreatment preparation included CT simulation, contrast-enhanced MRI for treatment planning, and individualised dosimetric planning.

The prescribed treatment course was completed in full and was generally well tolerated. Grade 1 acute skin toxicity according to the Radiation Therapy Oncology Group criteria was recorded, manifesting as localised erythema. No new neurological adverse events attributable to proton beam therapy were observed. After completing treatment, the patient was discharged with recommendations for continued follow-up by an oncologist and neurologist and surveillance contrast-enhanced MRI of the cervicothoracic spine.

## Radiological follow-up

**Figure 5:**
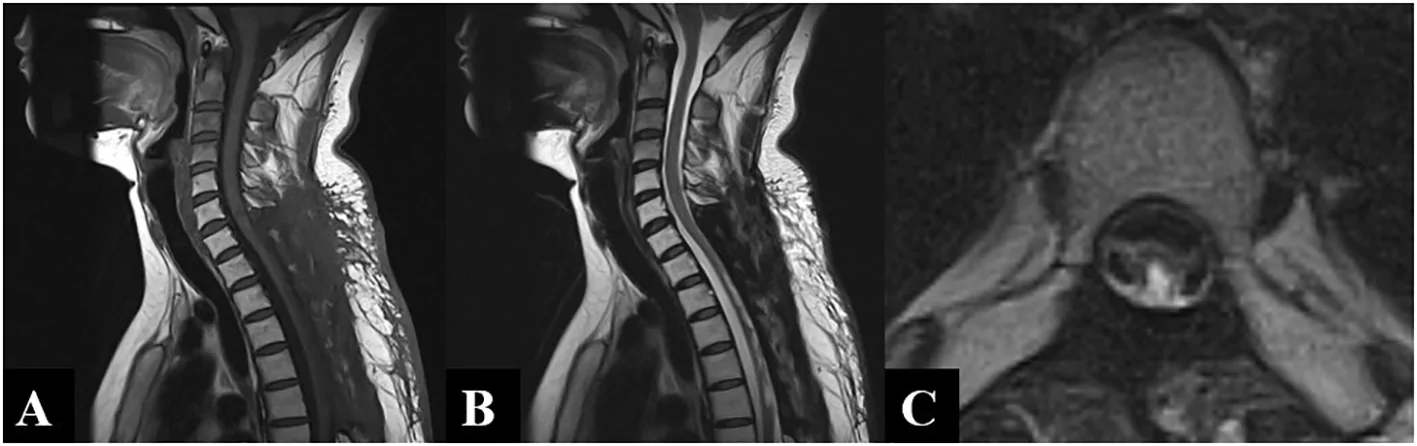
Six-month follow-up magnetic resonance imaging of the cervicothoracic spinal cord. **(A)** Sagittal T1-weighted image. **(B)** Sagittal T2-weighted image. **(C)** Axial T2-weighted image. Images demonstrate irregular spinal cord thinning and cystic–gliotic postoperative changes at T1–T5, without definite MRI evidence of disease progression at the time of examination.

Follow-up contrast-enhanced MRI of the cervical and upper thoracic spine was performed on 10 June 2026, approximately six months after surgery and three months after completion of proton beam therapy. Irregular thinning of the spinal cord and cystic–gliotic changes, predominantly involving its posterior aspect, were observed at the T1–T5 levels. No pathological contrast enhancement was identified. These findings were interpreted as stable postoperative changes, with no definite MRI evidence of disease progression at the time of examination (**Figure 5**).

Given the relatively short follow-up period, these MRI findings do not permit definitive conclusions regarding long-term local disease control. Continued clinical and radiological surveillance with serial contrast-enhanced MRI was recommended.

## Discussion

This case describes a rare long-segment intramedullary ependymoma of the cervicothoracic spinal cord that became clinically manifest during the third trimester of pregnancy and was diagnosed after delivery following rapid neurological deterioration. The disease resulted in severe spastic paraparesis, a defined sensory level, and sphincter dysfunction, necessitating microsurgical gross total resection. Spinal ependymomas diagnosed during pregnancy or the postpartum period have predominantly been reported as isolated cases and in small literature reviews. The present observation should therefore be regarded as an additional documented case rather than evidence that pregnancy directly caused tumour growth or accelerated its progression (5, 6, 8). The available evidence supports increased diagnostic vigilance but does not permit reliable conclusions regarding a pregnancy-specific incidence of ependymoma, hormonal stimulation of tumour growth, or an increased risk of recurrence.

Diagnostic delay in such cases is understandable but potentially harmful. Back pain is common during pregnancy, with systematic reviews reporting an overall prevalence of approximately 40.5% and a third-trimester prevalence of 47.8% (9). Early manifestations of spinal cord disease may consequently be misinterpreted as physiological musculoskeletal changes associated with pregnancy. However, progressive paraparesis, a defined sensory level, pyramidal signs, and urinary retention are not physiological manifestations of pregnancy and should raise concern for spinal cord pathology. In the present case, the first symptoms developed at approximately 30 weeks of gestation and were initially managed conservatively without neuroimaging or specialist neurological assessment. MRI was performed only after delivery, when marked neurological deterioration had occurred.

When objective signs of myelopathy are present, non-contrast MRI of the spine and spinal cord should not be delayed solely because of pregnancy. MRI does not involve ionising radiation and may be used during pregnancy when clinically justified. Gadolinium-based contrast agents should be reserved for situations in which contrast enhancement is expected to provide substantial diagnostic benefit and influence management or outcomes for the mother or foetus (33). In the present case, contrast-enhanced MRI was performed after delivery; therefore, the foetus was not exposed to a gadolinium-based contrast agent.

The neuroimaging findings were consistent with the typical appearance of a spinal ependymoma. Characteristic MRI features include a central intramedullary location, relatively well-defined margins, symmetrical fusiform expansion of the spinal cord, variable contrast enhancement, and possible cystic components, haemorrhage, or haemosiderin deposition (11–13). In this case, the tumour was centrally located, extended from C7 to T5, caused symmetrical fusiform expansion of the spinal cord, and demonstrated contrast enhancement, supporting ependymoma as the leading preoperative diagnosis.

The principal differential diagnoses included intramedullary astrocytoma, haemangioblastoma, and an inflammatory demyelinating lesion of the spinal cord. The absence of a pronounced infiltrative growth pattern, eccentric location, and asymmetrical spinal cord expansion made astrocytoma less likely. Haemangioblastoma was also considered unlikely because characteristic features, including prominent feeding or draining vessels with flow voids, a superficial or exophytic hypervascular component, and marked associated syringomyelia, were absent. The gradually progressive clinical course combined with a persistent contrast-enhancing mass causing spinal cord expansion was also less typical of longitudinally extensive transverse myelitis or tumefactive demyelination (12–14).

The timing of surgery for spinal cord tumours detected during pregnancy should be individualised according to the mother’s neurological status, gestational age, foetal viability, rate of symptom progression, and anticipated operative risk. In neurologically stable patients, surgery may in selected cases be postponed until after delivery. Progressive myelopathy, however, requires urgent multidisciplinary assessment involving neurosurgeons, obstetricians, anaesthetists, neuroradiologists, and neonatologists (5, 10, 15). In the present case, delivery had already occurred by the time the patient was referred to the specialised neurosurgical centre, and surgery was indicated because of severe progressive postpartum myelopathy with marked paraparesis and urinary retention.

Following multidisciplinary assessment, the indication for surgical treatment was confirmed. Brief preoperative preparation included anti-oedema therapy, assessment of the patient’s general and obstetric status, and ultrasonography of the abdominal and pelvic organs. This preparation was considered necessary because of the early postpartum period, the anticipated extent of surgery, and the need to exclude additional contraindications. Microsurgical tumour resection was performed after completion of the assessment.

Gross total resection is the principal surgical objective for spinal ependymomas when a safe dissection plane exists between the tumour and the surrounding spinal cord tissue (16–22). Adult surgical series indicate that the extent of resection and severity of the preoperative neurological deficit are among the most important determinants of local tumour control and functional recovery. In the present case, the relatively well-defined interface between the tumour and the spinal cord parenchyma permitted the development of a microsurgical dissection plane and gross total resection. The absence of residual contrast-enhancing tumour was confirmed on early postoperative MRI.

Intraoperative neurophysiological monitoring provides additional information regarding the functional integrity of spinal cord pathways during the resection of intramedullary tumours. Motor- and somatosensory-evoked potentials are commonly used, while D-wave monitoring, when technically available, may provide additional prognostic information regarding long-term motor outcome (23–25). In the present case, initial motor responses were of low amplitude, potentially because of severe pre-existing spinal cord pathway dysfunction and the effects of neuromuscular blockade and anaesthetic agents. After the anaesthetic regimen was changed from sevoflurane to propofol, reproducible motor responses were obtained from the upper and lower extremities, with higher baseline amplitudes on the right. By the end of resection, motor responses remained stable in the right upper and lower extremities, whereas responses from the left upper extremity were markedly reduced and reproducible responses from the left lower extremity were absent. Despite these intraoperative changes, no postoperative worsening of the neurological deficit was observed.

During the early postoperative period, spastic paraparesis, sensory impairment below T1, and neurogenic bladder dysfunction characterised by urinary retention persisted. Nevertheless, lower-extremity motor function gradually improved. The patient was mobilised, was able to sit independently, and moved around the ward using a wheelchair. The urinary catheter remained in place at discharge. No cerebrospinal fluid leakage, surgical-site infection, or other postoperative complications were recorded. The patient was discharged for outpatient care on 1 January 2026, postoperative day 21, with recommendations for continued neurological rehabilitation and referral to a specialised oncology centre.

Histopathological examination demonstrated characteristic ependymal differentiation based on numerous perivascular pseudorosettes, GFAP expression, and dot-like cytoplasmic EMA immunoreactivity. Increased mitotic activity, microvascular proliferation, focal coagulative necrosis, and intratumoral haemorrhage were described as high-grade morphological features. The tumour was classified as ependymoma, NOS, CNS WHO grade 3 in the pathology report dated 18 December 2025.

The 2021 WHO Classification of Tumours of the Central Nervous System classifies ependymal tumours according to their anatomical location and molecular characteristics (26–28). Spinal ependymoma with MYCN amplification is recognised as a distinct rare molecular type associated with more aggressive clinical behaviour, an increased risk of dissemination, and an unfavourable prognosis (29–31). Molecular genetic testing performed on 14 July 2026 detected no IDH1 or IDH2 mutations and identified a heterozygous deletion of MYB. These findings did not permit assignment of the tumour to a specific molecular type under WHO CNS5. The clinical and diagnostic significance of an isolated heterozygous MYB deletion in this setting remains uncertain. Testing for MYCN amplification and genome-wide DNA methylation profiling was not performed; consequently, the NOS designation was retained because complete molecular classification was unavailable.

After surgery, the patient was referred to the National Scientific Cancer Centre, and the case was reviewed by a multidisciplinary tumour board on 26 January 2026. Based on the diagnosis of ependymoma, NOS, CNS WHO grade 3, adjuvant proton beam therapy to the postoperative tumour bed was recommended. The tumour location and its considerable craniocaudal extent were additionally considered during treatment planning. According to the EANO guidelines, postoperative radiotherapy at a dose of 45–54 Gy is recommended for grade 3 spinal ependymoma regardless of the extent of resection. Following gross total resection of a grade 2 tumour, observation is generally recommended, whereas local postoperative radiotherapy at a dose of 45–54 Gy is recommended after incomplete resection (32).

Between 28 January and 12 March 2026, the patient completed a full course of image-guided proton beam therapy to the postoperative tumour bed. A dose of 1.8 Gy per fraction was delivered to a total dose of 54 Gy. The prescribed course was completed in full. Grade 1 acute skin toxicity according to the Radiation Therapy Oncology Group criteria, manifesting as localised erythema, was recorded. No new neurological adverse events attributable to proton beam therapy were observed.

At approximately six months after surgery, partial neurological recovery was observed. Lower-extremity muscle strength improved from MRC grade 2/5 to 3–4/5, and functional status improved from grade IV to grade III on the modified McCormick scale. Partial recovery of bladder function was reported, although residual neurogenic dysfunction persisted. Follow-up contrast-enhanced MRI of the cervical and upper thoracic spine performed on 10 June 2026 showed irregular thinning of the spinal cord and cystic–gliotic changes, predominantly involving its posterior aspect, at the T1–T5 levels. No pathological contrast enhancement was identified. These findings were interpreted as stable postoperative changes with no definite MRI evidence of disease progression. However, the relatively short follow-up period is insufficient to assess long-term local control or the risk of late recurrence. Comparison with previously reported pregnancy-associated spinal ependymomas further highlights several distinctive features of the present case (**Table 2**). Fujii et al. described a similarly long-segment cervicothoracic ependymoma in a 28-year-old woman whose symptoms began at approximately 20 weeks of gestation and progressed to severe paraparesis, a defined sensory level, urinary retention, and bowel dysfunction by the time of delivery. Despite gross total resection on postpartum day 20, paraplegia and bladder and bowel dysfunction persisted at 1-year follow-up (5). In contrast, van der Hoeven et al. reported a C5–T1 ependymoma presenting only with severe nocturnal thoracic pain during pregnancy, without an objective motor deficit; complete recovery was achieved after postpartum tumour resection (34). These contrasting outcomes support the observation that the severity and duration of the preoperative neurological deficit may be more important determinants of functional recovery than tumour size or anatomical extent alone.

**Table 2:** Published cases of pregnancy-associated spinal ependymoma: diagnosis and surgical management.

Source	Age (years)	Diagnosis	Surgical treatment
Han et al., 2008	NR	Recurrent intramedullary ependymoma, C3–T2; histological grade NR	Tumour resection after preterm vaginal delivery at 33 weeks
Fujii et al., 2018	28	Long-segment intramedullary ependymoma, C3–T5/C4–T6, WHO grade 2	C3–T6 laminectomy and gross total resection on postpartum day 20
van der Hoeven et al., 2018	31	Intramedullary ependymoma, C5–T1, WHO grade 2	Postpartum microsurgical debulking; follow-up MRI indicated total resection
Bitterman et al., 2019	28	Thoracic spinal ependymoma, approximately T5–T8; histological grade NR	Emergency T5–T8 laminectomy and tumour resection during pregnancy
Thakar et al., 2020	21	Cellular intramedullary ependymoma, T10–T12, WHO grade 2	Near-total tumour resection during pregnancy
Butenschoen et al., 2021	34	Intramedullary ependymoma, T10–T11, WHO grade 2	T10–T11 hemilaminectomy and biopsy at 19 weeks, followed by gross total resection under MEP monitoring 1 week later
**Present case**	**29**	**Long-segment intramedullary ependymoma, NOS, C7–T5, CNS WHO grade 3**	**Postpartum C7–T5 laminectomy, midline myelotomy and microsurgical gross total resection with intraoperative neurophysiological monitoring**

The case described in this article is highlighted in bold.

Other reports illustrate the wide clinical spectrum and variable timing of surgical treatment. Bitterman et al. described thoracic pain beginning early in pregnancy that was initially attributed to pregnancy-related musculoskeletal changes and subsequently progressed to paraplegia requiring emergency tumour resection and intensive rehabilitation (15). Butenschoen et al. reported a T10–T11 grade 2 ependymoma diagnosed at 19 weeks of gestation in a patient with progressive genital hypoaesthesia and incomplete paraplegia. Gross total resection was successfully performed during pregnancy under motor-evoked potential monitoring, with improvement to only minor residual left-leg weakness at 5-year follow-up (35). Han et al. reported a recurrent C3–T2 intramedullary ependymoma managed by preterm vaginal delivery at 33 weeks followed by tumour resection (36). Collectively, these observations demonstrate that the timing of surgery must be individualised according to gestational age, the rate of neurological deterioration, foetal viability, and the anticipated risk of irreversible spinal cord dysfunction.

The present case involved a long-segment C7–T5 cervicothoracic tumour that became clinically manifest at approximately 30 weeks of gestation and was followed by rapid postpartum deterioration. At admission, the patient had severe spastic paraparesis with predominantly MRC grade 2/5 lower-extremity strength, sensory impairment below T1, urinary retention with loss of bladder-filling sensation, and a modified McCormick grade of IV. Unlike most previously reported pregnancy-associated spinal ependymomas, which were classified as WHO grade 2 or lacked detailed histological grading, the present tumour was diagnosed as ependymoma, NOS, CNS WHO grade 3. The patient also received adjuvant proton beam therapy to the postoperative tumour bed at a total dose of 54 Gy, a treatment approach not described in the most closely comparable published cases.

Despite the severity of the preoperative myelopathy, partial neurological recovery was documented at approximately 6 months: lower-extremity strength improved to MRC grade 3–4/5, modified McCormick grade improved from IV to III, and partial recovery of bladder function was observed. Conversely, Thakar et al. reported a grade 2 conus ependymoma that developed a fatal holocord recurrence within 6 months of near-total resection (6). This marked variability in outcome underscores the limitations of histological grade alone for predicting biological behaviour and supports comprehensive molecular characterisation and prolonged contrast-enhanced surveillance. Nevertheless, the relatively short follow-up in the present case precludes conclusions regarding long-term local control, and the current findings should be interpreted only as evidence of early clinical improvement and absence of definite radiological progression.

According to the EANO guidelines, craniospinal MRI and cerebrospinal fluid cytology performed no earlier than 2–3 weeks after surgery should be considered after resection of a newly diagnosed ependymoma because of the potential for dissemination through cerebrospinal fluid pathways (32). In the present case, complete craniospinal staging and cerebrospinal fluid cytology were not documented. Given the CNS WHO grade 3 diagnosis and incomplete molecular characterisation, the absence of these assessments limits evaluation of the risk of leptomeningeal dissemination. They should be considered as part of continued oncological surveillance.

The principal practical lesson from this case is that the high prevalence of back pain during pregnancy should not obscure objective signs of spinal cord dysfunction. Progressive paresis, a defined sensory level, pyramidal signs, or sphincter dysfunction warrant urgent neurological assessment, MRI of the spine and spinal cord, and referral to a specialised centre experienced in the surgical management of intramedullary tumours.

The strengths of this report include detailed clinical documentation, a chronological description of diagnostic and therapeutic management, multidisciplinary assessment, preoperative and early postoperative contrast-enhanced MRI, microsurgical gross total resection, intraoperative neurophysiological monitoring, documented proton beam therapy, and approximately six months of clinical and contrast-enhanced MRI follow-up. The main limitations are the single-case design, short follow-up period, and absence of a complete integrated molecular diagnosis, including MYCN amplification testing and genome-wide DNA methylation profiling. The absence of documented craniospinal staging and cerebrospinal fluid cytology represents an additional limitation. Finally, this case cannot establish a causal relationship between pregnancy and tumour growth, progression, or biological behaviour.

Because spinal ependymomas may recur late and initially remain asymptomatic, long-term clinical surveillance with regular contrast-enhanced MRI is required. The findings obtained at approximately six months should be interpreted only as evidence of early local disease control and not as definitive confirmation of cure.

## Conclusion

An intramedullary spinal cord lesion should be considered in pregnant or postpartum patients who develop progressive neurological deficits, particularly when symptoms persist or worsen after delivery. Progressive motor weakness, a defined sensory level, pyramidal signs, or sphincter dysfunction should prompt urgent neurological assessment and spinal MRI. This case highlights the importance of early recognition, multidisciplinary management, maximal safe microsurgical resection with intraoperative neurophysiological monitoring, molecular characterisation when available, and long-term surveillance with contrast-enhanced MRI. However, this single observation does not establish a causal relationship between pregnancy and ependymoma growth or progression.

## Data Availability

The original contributions presented in the study are included in the article/**Supplementary Material**. Further inquiries can be directed to the corresponding author.
