# Supplementary material for: Case Report: Postpartum diagnosis and microsurgical management of a cervicothoracic intramedullary spinal ependymoma clinically manifesting during the third trimester of pregnancy
Source: Front Oncol. 2026 Sep 8;16:1926208. doi: 10.3389/fonc.2026.1926208 (PMC13593514; doi:10.3389/fonc.2026.1926208)
Supplement: Supplementary file 1 [file DataSheet1.pdf]

CARE case report guidelines

CARE Checklist of information to include when writing a case report

(cc) BY-NC-ND

Topic	Item No	Checklist item description	Reported on Page Number/Line Number	Reported on Section/Paragraph
Title	1	The diagnosis or intervention of primary focus followed by the words "case report"	p. 1	Title page / Title
Key Words	2	2 to 5 key words that identify diagnoses or interventions in this case report, including "case report"	p. 2	Keywords
Abstract (Structured summary)	3a	Background: state what is known and unknown; why the case report is unique and what it adds to existing literature.	pp. 1–2	Abstract / Background
	3b	Case Description: describe the patient's demographic details, main symptoms, history, important clinical findings, the main diagnosis, interventions, outcomes and follow-ups.	pp. 1–2	Abstract / Case presentation
	3c	Conclusions: summarize the main take-away lesson, clinical impact and potential implications.	p. 2	Abstract / Conclusion
Introduction	4	One or two paragraphs summarizing why this case is unique **(may include references)**	p. 3	Introduction, paragraphs 1–3
Patient Information	5a	De-identified patient specific information	p. 3	Case description / Clinical presentation and timeline, paragraph 1
	5b	Primary concerns and symptoms of the patient	p. 3	Case description / Clinical presentation and timeline, paragraphs 1–2
	5c	Medical, family, and psycho-social history including relevant genetic information	p. 3	Case description / Clinical presentation and timeline, paragraph 1; relevant family, psycho-social and genetic history not reported
	5d	Relevant past interventions with outcomes	pp. 3–4	Case description / Clinical presentation and timeline; Table 1
Clinical Findings	6	Describe significant physical examination (PE) and important clinical findings	p. 4	Diagnostic assessment, paragraph 1
Timeline	7	Historical and current information from this episode of care organized as a timeline	p. 4	Table 1. Clinical timeline of the episode of care
Diagnostic Assessment	8a	Diagnostic testing (such as PE, laboratory testing, imaging, surveys).	pp. 4–5, 7–8	Diagnostic assessment; Histopathological findings; postoperative MRI findings
	8b	Diagnostic challenges (such as access to testing, financial, or cultural)	pp. 3, 5, 10	Case description and Discussion: pregnancy-related diagnostic delay; MRI performed postpartum; no fetal gadolinium exposure
	8c	Diagnosis (including other diagnoses considered)	pp. 4–5, 7	Diagnostic assessment; differential diagnosis; Histopathological findings
	8d	Prognosis (such as staging in oncology) where applicable	pp. 7, 9, 11–12	Histopathological findings: ependymoma, NOS, CNS WHO grade 3; Follow-up and outcomes; Discussion/limitations
Therapeutic Intervention	9a	Types of therapeutic intervention (such as pharmacologic, surgical, preventive, self-care)	p. 6	Therapeutic intervention, paragraph 1
	9b	Administration of therapeutic intervention (such as dosage, strength, duration)	p. 6	Therapeutic intervention, paragraphs 1–4

	9c	Changes in therapeutic intervention (with rationale)	pp. 8–9	Follow-up and outcomes: rehabilitation; individualized decision not to administer adjuvant radiotherapy and surveillance strategy
Follow-up and Outcomes	10a	Clinician and patient-assessed outcomes (if available)	pp. 8–9	Follow-up and outcomes; Patient perspective
	10b	Important follow-up diagnostic and other test results	pp. 8–9	Follow-up and outcomes: postoperative day 1 MRI and 6-month follow-up MRI
	10c	Intervention adherence and tolerability (How was this assessed?)	pp. 8–9	Follow-up and outcomes: postoperative course, rehabilitation discharge and surveillance; tolerability described by absence of complications
	10d	Adverse and unanticipated events	pp. 6, 8–9	Therapeutic intervention and Follow-up and outcomes: no IONM deterioration, no CSF leakage, wound infection, postoperative complication, or radiological progression
Discussion	11a	A scientific discussion of the strengths AND limitations associated with this case report	p. 12	Discussion, strengths and limitations paragraph
	11b	Discussion of the relevant medical literature **with references**	pp. 10–12	Discussion, literature review paragraphs with references
	11c	The scientific rationale for any conclusions (including assessment of possible causes)	pp. 10–12	Discussion: diagnostic, surgical, histopathological and adjuvant-treatment rationale
	11d	The primary “take-away” lessons of this case report (without references) in a one paragraph conclusion	p. 12	Conclusion; final take-away lesson paragraph
Patient Perspective	12	The patient should share their perspective in one to two paragraphs on the treatment(s) they received	p. 9	Patient perspective
Informed Consent	13	Did the patient give informed consent? Please provide if requested	Yes; p. 12	Ethics statement / Written informed consent for publication
